# Characterization of the complete mitochondrial genomes and phylogenetic analysis of the two *Luciogobius* species (Perciformes, Gobionellinae) from Korea

**DOI:** 10.1080/23802359.2018.1522980

**Published:** 2018-10-29

**Authors:** Jumin Jun, Seung-Ho Choi, Hee Young Kim

**Affiliations:** a Animal Resources Division, National Institute of Biological Resources, Incheon, Republic of Korea;; b Division of EcoScience, Ewha Womans University, Seoul, Republic of Korea;; c SOKN Institute of Ecology and Conservation, Inc., Gyounggi-do, Republic of Korea

**Keywords:** Perciformes, Gobionellinae, mitochondrial genome, *Luciogobius elongatus*, *Luciogobius grandis*

## Abstract

The complete mitochondrial genomes of *Luciogobius grandis and Luciogobius elongatus* (Perciformes, Gobionellinae) living on rocky coasts in South Korea are reported here for the first time. The mitogenomes of *L. grandis* and *L. elongatus* have total lengths of 16,477 bp and 16,486 bp, respectively, and include 13 PCGs, small and large rRNAs, a control region, and 22 tRNAs. Most of the genes are encoded on the heavy strand except for nine genes. Compared with other Gobionellinae species, the PCGs of the two *Luciogobius* species have a conserved gene order. These data provide useful molecular information for phylogenetic studies concerning *Luciogobius* species and related species.

*Luciogobius,* a genus of the Gobionellinae, comprises 16 species and is mainly distributed in north-eastern Asia (Kanagawa et al. 2011). Seven *Luciogobius* species were reported in Korea (The National Institute of Biological Resources 2017), but data are lacking on their biological characteristics and genetic information. In this study, we first determined the complete mitochondrial genomes of the two *Luciogobius* species, *L. grandis* and *L. elongatus*.

The specimens were collected from Jangmok-myeon, Geoje-si, Gyoungsangnam-do on the southern coast of South Korea (deposited in National Institute of Biological Resources, accession numbers SH130607-3B (*L. grandis*) and SH130607-3A (*L. elongatus*)). PCR primers based on short fragment sequences of *COX1* (Ward et al. 2005) were designed to amplify and sequence the complete mitochondrial DNA.

The total lengths of the mitogenomes of *L. grandis* and *L. elongatus* were 16,477 bp and 16,486 bp, respectively, with a genome size similar to other gobies. The annotated mitochondrial genome sequences were submitted to GenBank with accession numbers MH682216 (*L. grandis*) and MH682217 (*L. elongatus*).

The mitochondrial genome of both species contains large and small rRNAs, 22 tRNAs, a control region, and 13 protein coding genes. The tRNAs and PCGs were predicted using tRNAscan-SE 1.21 (Schattner et al. 2005) and ORFfinder (https://www.ncbi.nlm.nih.gov/orffinder/). The A/T contents are 58.2% in *L. grandis* and 57.8% in *L. elongatus*. Most genes are encoded on the heavy strand, except for one PCG (*ND6*) and eight tRNA genes (*tRNA*^Pro^, *tRNA*^Glu^, *tRNA*^Ser^, *tRNA*^Tyr^, *tRNA*^Cys^, *tRNA*^Asn^, *tRNA*^Ala^, and *tRNA*^Gln^). All the PCG genes start with the orthodox ATG start codon, whereas *COX1* gene starts with GTG. A GTG codon has also been reported as a start codon of *COX1* gene in other *Luciogobius* species (Jin et al. 2013; Yu et al. 2015). In the mitogenome of *L. grandis*, six PCGs share the termination codon TAA (*COX1, ATP8, ATP6, ND4L, ND5*, and *ND6*) and three PCGs have TAG as a termination codon (*ND1, ND2*, and *ND3*). In *L. elongatus*, seven PCGs use TAA as a stop codon (*ND1, ND2, COX1, ATP8, ATP6, ND4L*, and *ND5*) and two PCGs have TAA (*ND3* and *ND6*). In both of the two species, four PCGs (*COX2, COX3, ND4*, and *CYTB*) have incomplete codon T, which is common among vertebrate mitochondrial protein-coding genes (Ojala et al. 1981). The gene composition and order of *L. grandis* and *L. elongatus* were identical to those of other Gobionellinae including the genus *Luciogobius* (Jin et al. 2013; Yu et al. 2015). Most of the tRNA genes can be folded into typical cloverleaf secondary structures, except for tRNA^Ser(UGA)^, which lacks a D arm in the mitogenomes of *L. grandis* and *L. elongatus*. The three tRNA clusters (IQM, WANCY, and HSL) are well conserved in the two species as those of typical vertebrate mitogenomes (Jin et al. 2013).

The complete mitochondrial genomes of *L. grandis* and *L. elongatus* and other Gobionellinae species were used to reconstruct a maximum likelihood tree with 1000 bootstrap replicates (Figure 1). The phylogenetic analyses were performed using RAxML (v8.2.10) (Stamatakis 2014) on CIPRES website.

**Figure 1.**
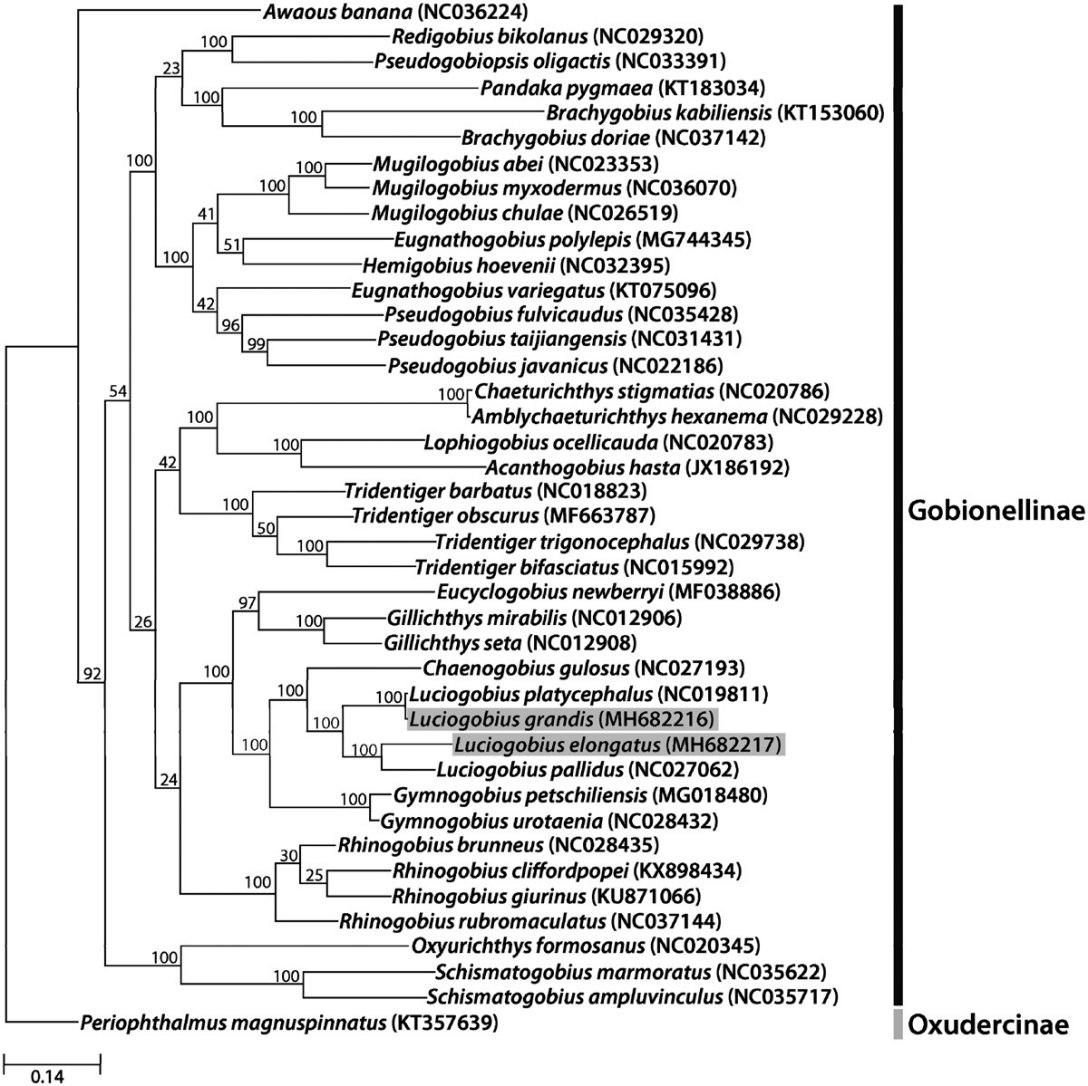
The molecular phylogenetic tree of the two *Lucigobius* species and other related species in Gobionellinae based on mitochondrial 13 protein-coding gene sequences. The complete mitochondrial genomes were obtained from GenBank and the accession numbers of the sequences are indicated in parentheses after scientific name of each species. The phylogenetic tree constructed by a maximum-likelihood method with 1,000 bootstrap replicates.
